# Usage, knowledge and perception of the ketogenic diet and associated factors in Saudi adults: A cross-sectional study

**DOI:** 10.1097/MD.0000000000037063

**Published:** 2024-02-09

**Authors:** Ameerah A. Alhassani, Essra A. Noorwali

**Affiliations:** a Clinical Nutrition Department, Faculty of Applied Medical Sciences, Umm Al-Qura University, Makkah 21955, Saudi Arabia

**Keywords:** ketogenic diet, obesity, Saudi Arabia, weight loss

## Abstract

The ketogenic diet (KD) is a popular weight management method. However, knowledge, perceptions, and use of KD have not been studied in the Saudi population. This cross-sectional study aims to assess the knowledge, perceptions, and use of the KD in Saudi Arabia, to compare medical and nonmedical students in their awareness of KD, and to determine factors associated with knowledge and perception of KD. A total of 1071 Saudis aged ≥ 18 years old were included. Participants were excluded if they were younger than 18, non-Saudi, pregnant, breastfeeding, had chronic illnesses, were using any medication, or were diagnosed with psychiatric conditions. Knowledge, perception, and use of KD were collected between 2021 and 2022 in Saudi Arabia using a prevalidated tool and its scoring protocol; higher scores indicated higher level of knowledge or perception. Among Saudi adults, 24% (*n* = 253) and 55% of them (*n* = 138/253) followed the KD for 1 month or less, respectively. The KD knowledge score was mean ± SD: 7.95 ± 3.97 and half of the participants (49.5%) had a low knowledge level. Students had a significantly lower knowledge score (mean ± SD: 7.69 ± 3.85) compared to nonstudents (mean ± SD: 8.68 ± 4.19). Students in medical majors had a higher knowledge score compared to nonmedical major students (*P* < .001). The KD perception score was mean ± SD: 28.74 ± 4.83 and 76% of the participants had moderate perception level. Males had a significantly higher perception score compared to females (*P* < .001). Students in medical majors had a lower perception score compared to nonmedical major students. Age and body mass index had a significantly weak positive correlation with knowledge and perception scores. Half of the sample had low knowledge and moderate level of perception about KD. Students with medical majors had higher knowledge but lower perception scores compared to nonmedical majors. Dieticians may measure the knowledge and perception of the KD diet in outpatient clinics to lose weight. Educational institutions may increase students’ knowledge and perception of the KD in all majors. Future experimental studies examining the efficacy of the KD are needed to provide recommendations of the KD as a strategy for weight loss.

## 1. Introduction

Noncommunicable diseases (NCDs) are chronic diseases such as diabetes, cardiovascular diseases (CVDs), chronic respiratory diseases, and cancers. NCDs are caused by a combination of genetic, environmental, physiological and behavioral factors and kill 41 million people each year, equivalent to 71% of all deaths globally.^[1]^ Overweight and obesity are leading risk factors contributing to the disease burden of NCDs. 69% of Saudi adults are overweight or obese and obesity is the second risk factor for death in Saudis.^[2]^ High body mass index (BMI) is a diagnostic criteria for overweight and obesity and is one of the main reasons for increasing Saudi Arabia’s morbidity and disease burden.^[3]^ A healthy body weight reduces the risk of morbidity and mortality.^[4]^ A balanced diet and regular exercise are the 2 most basic criteria for a healthy body weight.^[5]^ There are a variety of carefully planned diet patterns that people can adopt to lose weight with the help of a trained professional, but a lack of understanding among them exacerbates the issue. Therefore, studying the knowledge of diets used to lose weight and factors associated with them is significant.

The ketogenic diet (KD) requires a firm restriction of carbohydrates and allows liberal ingestion of fats.^[6]^ The KD is widely used to treat a variety of metabolic disorders as well as weight loss.^[7]^ Several studies indicated that KD has several benefits: weight loss, lowers fasting glucose and insulin levels, fewer exercise-induced oxidative stress and inflammation, increased memory and mood, decreased fat oxidation, and glycogen sparing.^[8]^ On the other hand, the most common and mild short-term side effects of the KD include nausea, vomiting, headache, exhaustion, dizziness, insomnia, trouble with exercise tolerance, and constipation, which is often referred to as the “keto flu.”^[9]^ In a few days to a few weeks, these signs will go away. Any of these effects can be alleviated by ensuring sufficient fluid and electrolyte intake.^[10]^ Kidney stones and vitamin and mineral deficiencies are all long-term side effects.^[9,10]^

Few studies have assessed the awareness and knowledge of individuals on the KD. A study conducted by Umair et al, among medical students in Pakistan found that medical students had a strong understanding of the medicinal applications, side effects, and health benefits of the KD. The most significant factor in following a KD was found to be weight loss.^[11]^ Furthermore, another study conducted by Alexandria M. D’Agostino among college students at Midwestern University in the USA found that awareness and understanding of the KD are inconsistent among male college students and are unlikely to affect KD usage due to other factors such as lifestyle habits and values that are more likely to affect KD participation.^[12]^

In Saudi Arabia, studies have been conducted to assess the utilization and efficacy of the KD in adults with chronic diseases^[13]^ and children with epilepsy.^[14,15]^ A recent study was conducted among Saudi students in Jazan that assessed the knowledge and perception of the KD.^[16]^ However, several limitations were observable including no comparison was conducted between health and nonhealth sciences and the factors associated with the knowledge and perception of the KD were not studied. Therefore, this study aims to assess the knowledge, perception, and use of KD among healthy Saudi adults, to compare knowledge, perception, and use of the KD between medical and nonmedical students and to study factors associated with the knowledge and perception of the KD.

## 2. Material and methods

### 2.1. Study design and participants

A cross-sectional online survey was created. Ethical approval was obtained on September, 7, 2021 from the biomedical research ethics committee at Umm Al-Qura University, approval No. (HAPO-02-K-012-2021-09-748). All methods were conducted in accordance with the ethical standards of the declaration of Helsinki. The first page of the questionnaire contained an informed consent (supplementary material 1, http://links.lww.com/MD/L345) that clearly states the aim of the study and how the participant data will be handled. Informed consent to participate statement was obtained by completing this questionnaire. Researchers collected questionnaires mainly from medical and nonmedical students, but also the general population were included in the study. Exclusion criteria: participants younger than 18 years, non-Saudis, pregnant, breastfeeding, having chronic illnesses, using any medication, or those with diagnosed psychiatric conditions. No missing data was available because submitting the questionnaire required complete information. All selected participants gave their consent as a prerequisite to enrollment.

Sample size calculation was conducted using https://www.calculator.net/sample-size-calculator.html. The minimum number of necessary samples to meet the desired statistical constraints is 385 participants or more are needed to have a confidence level of 95% that the real value is within ± 5% of the measured/surveyed value.

Participants received the questionnaire link on Google Forms via social media platforms such as Twitter and WhatsApp between 2021 and 2022 in Saudi Arabia. The survey was distributed through open channels, specifically social media platforms. As a result, it was not possible to determine the precise number of individuals who viewed or had access to the survey. Consequently, calculating the response rate in the traditional sense, which requires knowing the total number of potential participants, is not feasible. After consenting and agreeing to participate, the questionnaire collected sociodemographic, self-reported anthropometrics, knowledge, perceptions, and use of the KD.

### 2.2. Assessment of KD knowledge and perception scores

A prevalidated questionnaire was used to assess the knowledge and perception of the KD.^[12,17]^ The questionnaire was used in 2 languages (Arabic and English). It was translated and revised from English to Arabic by 2 researchers (AH and EN) using a forward–backward translation method. To maintain the accuracy of the translated statements, we put more emphasis on conceptional translation than word-by-word translation. Prior to use in this study, the questionnaire was initially administered to 10 bilingual subjects, who completed both the Arabic and English version to determine the test–retest reliability. Usage, knowledge, and perception scores were independent of the version. Eighteen questions about the KD’s development, composition, fuel sources, ketone bodies, type of fat, weight loss, blood cholesterol, complications, and research were used to test knowledge. Participants’ answers were compared to a predefined correction key. The knowledge responses were categorized based on a total score for all knowledge questions (*K* = 18), with the lowest score being “zero” (answering all questions incorrectly or declaring I don’t know) and the highest score being 18. Nine questions were used to assess the participants’ perception of the KD. The minimum score was 9 (answered strongly disagree to all of the questions) and the maximum score was 45, which was calculated by adding replies to all of the perceptual items (*K* = 9). Answers to the perception questions were given either positive or negative perception.^[17]^

Three perception questions were reverse coded meaning if participants answered strongly agree they were given negative values. The questions were: I believe the KD guidelines, in my opinion, are too high in fat and low in protein and carbohydrates; I believe the KD is risky (it raises a person’s risk of diseases like cardiovascular disease and diabetes); and I believe people on the KD are at risk of nutritional deficiencies.

### 2.3. Statistical analysis

To assess Saudi population’s knowledge, perceptions, and use of the KD, data analysis was performed using statistical package for the social sciences, SPSS 23rd version. Frequency and percentages were used to display categorical variables. Minimum, maximum, mean, and standard deviation were used to present numerical variables. Independent *t* test and ANOVA test were both utilized to determine factors associated with perception and knowledge toward the KD. Pearson correlation was also used to test the presence of correlation between numerical variables. The level of significance was set at 0.05. In addition, Tukey post hoc test was used to determine the significance of differences in group. To study the factors associated with knowledge toward KD and to compare between medical and nonmedical students in their knowledge and perception of the KD, participants were categorized to medical and nonmedical fields (supplementary material 2, http://links.lww.com/MD/L347).

## 3. Results

### 3.1. Participants’ demographics and experience with the KD diet

A total of 1216 participants completed the questionnaire. Twelve participants were excluded because their age was < 18 years. One hundred thirty-three participants were excluded because they were non-Saudis. A total of 1071 participants were included in this study all aged ≥ 18 years and living in Saudi Arabia. Table 1 shows the sociodemographic profile of the participants and their experience with the KD diet. The mean age of the participants was mean ± SD: 26.19 ± 8.97. The mean BMI was mean ± SD: 25.64 ± 8.48. Among the participants 790 (73.8%) were students.

**Table 1:** Sociodemographic characteristics and diet experience of Saudi participants (*n* = 1071)

Demographical characteristics	Mean	SD
Age	26.19	8.97
BMI	25.64	8.48

	*n*	%
Sex		
Male	239	22.30
Female	832	77.70
Student		
Yes	790	73.80
No	281	26.20
University/college major		
Medical	250	23.30
nonmedical	540	50.40
Not a student	281	26.20
Course level		
Diploma	24	2.20
Bachelor’s first	151	14.10
Bachelor’s second and above	513	47.90
Master	69	6.40
PhD	26	2.40
Others	7	0.70
Not s student	281	26.20
Participants’ experience with dieting		
	***n***	**%**
Have you followed a fad diet before? (a short-term weight-loss regimen like the cabbage diet, detox diet, etc)?		
Yes	22	2.1
No	1049	97.9
Do you know what the KD is?		
Yes	1029	96.1
No	42	3.9
Do you follow the KD now?		
Yes	5	0.5
No	818	76.4
No, but I have tried before	248	23.2
If you chose (yes) that you followed the KD, how long did you follow the KD? (*n* = 253)		
1–4 weeks and less (1 month and less)	138	54.5
5–12 weeks (1–3 months)	54	21.3
13–26 weeks (3–6 months)	15	5.9
More than 26 weeks (more than 6 months)	17	6.7
I’m not sure	29	11.4
Never followed a ketogenic diet	818 from 1071	76.40
Participants report on outcomes of following KD (*n* = 253)		
Weight loss and weight kept off	75	29.6
Weight loss but gained weight back	138	54.5
No change	18	7.1
Other	22	8.6

BMI = body mass index, KD = ketogenic diet, *n*; number, SD = standard deviation

Only 22 (2.1%) of the participants reported following a fad diet before. A total of 1029 (96.1%) of the participants reported knowing what the KD is. About 248 (23.2%) of the participants reported that they had previously tried following a KD, whereas 818 (76.4%) reported they never followed a KD.

For those who currently/previously followed a KD (*n* = 253), *n* = 138/253 (54.5%) of the participants reported following it for 1–4 weeks and *n* = 138/253 (54.4%) of the participants reported losing weight and gaining it back.

### 3.2. Participants’ knowledge and perception toward the KD

Table 2 demonstrates the assessment of participant’s knowledge toward the KD. The minimum knowledge score was 0, the maximum was 16, and the mean was 7.95 SD ± 3.97. Almost half of the participants *n* = 530 (49.5%) of the participants had a low knowledge level. Table 3 shows the assessment of participant’s perception toward the KD. The minimum perception score was 12, and the maximum was 44 (mean ± SD: 28.74 ± 4.83) and 814 of the participants (76%) had moderate perception level. About 45% of the participants strongly agree that the KD should only be recommended to individuals by a physician/dietitian and under their supervision.

**Table 2:** Knowledge of KD in Saudi adults (*n* = 1071)

Question	*n*	%
The KD was created as a clinical treatment for epilepsy patients		
True (correct answer)	386	36.00
False	103	9.60
I don’t know	582	54.30
The KD is a low-carbohydrate, high-fat, high-protein diet		
True	803	75.00
False (correct answer)	128	12.00
I don’t know	140	13.10
The KD helps to encourage the body to burn ketone bodies instead of glucose for energy		
True (correct answer)	707	66.00
False	79	7.40
I don’t know	285	26.60
Carbohydrates are the most common dietary source of ketone bodies		
True	204	19.00
False (correct answer)	560	52.30
I don’t know	307	28.70
The KD promotes the oxidation and utilization of fatty acids		
True (correct answer)	662	61.80
False	35	3.30
I don’t know	374	34.90
Ketosis is a metabolic condition in which the body uses ketone bodies as its primary fuel source		
True (correct answer)	529	49.40
False	71	6.60
I don’t know	471	44.00
As an energy source, the brain can only use glucose and ketone bodies		
True (correct answer)	368	34.40
False	242	22.60
I don’t know	461	43.00
In extreme cases of fasting, ketone bodies are used as a fuel source		
True (correct answer)	645	60.20
False	60	5.60
I don’t know	366	34.20
Without calorie restriction, the KD attempts to imitate a fasted metabolic state		
True	392	36.60
False (correct answer)	64	6.00
I don’t know	615	57.40
On the KD, the type of fat eaten (saturated, monounsaturated, polyunsaturated, and trans fats) makes no difference		
True	168	15.70
False (correct answer)	558	52.10
I don’t know	345	32.20
The KD comes in different types		
True (correct answer)	545	50.90
False	149	13.90
I don’t know	377	35.20
The KD aids in the improvement of hunger signals (makes you feel fuller and endless hungry)		
True (correct answer)	724	67.60
False	71	6.60
I don’t know	276	25.80
Weight loss is not induced by the KD		
True	52	4.90
False (correct answer)	857	80.00
I don’t know	162	15.10
The KD has been shown to have no effect on blood sugar levels		
True	136	12.70
False (correct answer)	486	45.40
I don’t know	449	41.90
The KD aids in the reduction of blood cholesterol levels (increases HDL-cholesterol and lowers LDL-cholesterol)		
True (correct answer)	425	39.70
False	116	10.80
I don’t know	530	49.50
Nutritional ketosis is described as a ketone body concentration in the blood of 1–3 mmol/dL		
True (correct answer)	203	19.00
False	32	3.00
I don’t know	836	78.10
An upset stomach, nausea, or vomiting are not common side effects of the KD		
True (correct answer)	202	18.90
False	408	38.10
I don’t know	461	43.00
The KD has been the subject of several long-term studies		
True (correct answer)	469	43.80
False	90	8.40
I don’t know	512	47.80
Knowledge score of KD (minimum possible score = 0, maximum possible score = 18)		
Low knowledge level (<50% of total score) (score of 8 and less)	530	49.5
Moderate knowledge level (between 50% and 75% of total score) (score between 9 and 13)	472	44.1
High knowledge level (higher than 75% of total score) (score of 14 and higher)	69	6.4
Minimum score	0	
Maximum score	16	
Mean	7.95	
Standard deviation	3.97	

KD = ketogenic diet, *n* = number

**Table 3:** Perception of KD in Saudi adults (*n* = 1071)

Question	*n*	%
I believe the KD is a common weight-loss strategy today		
Strongly disagree	22	2.10
Disagree	35	3.30
Neutral	147	13.70
Agree	408	38.10
Strongly agree (positive)	459	42.90
I believe that following the KD ensures weight loss		
Strongly disagree	26	2.40
Disagree	67	6.30
Neutral	259	24.20
Agree	421	39.30
Strongly agree (positive)	298	27.80
I believe the KD guidelines, in my opinion, are too high in fat and low in protein and carbohydrates		
Strongly disagree	69	6.40
Disagree	260	24.30
Neutral	349	32.60
Agree	230	21.50
Strongly agree (negative)	163	15.20
I believe that the health benefits of the KD outweigh the risks		
Strongly disagree	101	9.40
Disagree	215	20.10
Neutral	378	35.30
Agree	222	20.70
Strongly agree (positive)	155	14.50
I believe the KD is easy to follow		
Strongly disagree	154	14.40
Disagree	295	27.50
Neutral	257	24.00
Agree	258	24.10
Strongly agree (positive)	107	10.00
I believe the KD has no negative side effects and is suitable for anyone who desires to follow it for the rest of their lives		
Strongly disagree	316	29.50
Disagree	349	32.60
Neutral	241	22.50
Agree	109	10.20
Strongly agree (positive)	56	5.20
I believe the KD should only be recommended to individuals by a physician/dietitian and under their supervision		
Strongly disagree	17	1.60
Disagree	66	6.20
Neutral	220	20.50
Agree	288	26.90
Strongly agree (positive)	480	44.80
I believe the KD is risky (it raises a person’s risk of diseases like cardiovascular disease and diabetes)		
Strongly disagree	90	8.40
Disagree	197	18.50
Neutral	456	42.60
Agree	197	18.40
Strongly agree (negative)	130	12.10
I believe people on the KD are at risk of nutritional deficiencies		
Strongly disagree	55	5.10
Disagree	172	16.10
Neutral	352	32.90
Agree	287	26.80
Strongly agree (negative)	205	19.10
Perception score (minimum possible score = 9, maximum possible score = 45)		
Low perception level	100	9.3
Moderate perception level	814	76
High perception level	157	14.7
Minimum score	12	
Maximum score	44	
Mean	28.74	
Standard deviation	4.83	

KD: ketogenic diet

### 3.3. Factors associated with knowledge toward the KD

Table 4 presents the factors associated with knowledge toward the KD. Being a student was significantly associated with the knowledge score (*P* < .001), students had a significantly lower knowledge score (mean ± SD: 7.69 ± 3.85) compared to nonstudents (mean ± SD: 8.68 ± 4.19). Students’ major was also significantly associated with knowledge score (*P* < .001), students with medical majors had a significantly higher knowledge scores (mean ± SD: 9.1 ± 3.32) compared to nonmedical major students (mean ± SD: 7.04 ± 3.91). Course level was also significantly associated with knowledge score (*P* = .014). Tukey post hoc revealed that those who are taking a master degree had a significantly higher mean of knowledge compared to students in their second/third bachelor’s degree or in a PhD degree (*P* < .05). Moreover, participants who previously followed a fad diet had a significantly higher knowledge score toward the KD compared to participants who did not (*P* < .001) (mean ± SD: 10 ± 2.83 vs 7.91 ± 3.98). Following a KD was also significantly associated with knowledge toward KD (*P* < .001). Tukey post hoc test revealed that participants who previously followed a KD had significantly higher knowledge compared to participants who never did (*P* < .05). Knowledge score was also significantly associated with perception level (*P* < .001). Tukey post hoc test revealed that participants with high perception had a significantly higher knowledge score compared to participants with low or moderate perception (*P* < .05). Both age and BMI had a significant weak positive correlation with knowledge score (*P* < .001, and *P* = .002 respectively).

**Table 4:** Factors associated with knowledge toward KD in Saudi adults (*n* = 1071)

Factor	Knowledge score		*P* value
	Mean	Standard deviation	
Sex			
Male	8.19	4.22	0.299
Female	7.89	3.89	
Student			
Yes	7.69	3.85	<0.001*
No	8.68	4.19	
University/college major			
Medical	9.10	3.32	<0.001*
nonmedical	7.04	3.91	
Course level			
Diploma	7.04	3.94	0.007*
Bachelor’s first	7.91	3.51	
Bachelor’s second and above	7.54	3.85	
Master	9.06	4.08	
PhD	6.31	3.91	
Have you followed a fad diet before? (a short-term weight-loss regimen like the cabbage diet, detox diet, etc)			
Yes	10	2.83	<0.001*
No	7.91	3.98	
Do you follow the ketogenic diet now?			
Yes	10.60	2.19	<0.001*
No	7.49	4.07	
No, but I have tried before	9.43	3.20	
Perception level			
Low perception level (what is the score of low)	7.68	3.22	<0.001*
Moderate perception level	7.51	4.00	
High perception level	10.42	3.29	
Age correlation with knowledge score			
*P* value		<0.001*	
Pearson correlation		0.106	
BMI correlation with knowledge score			
*P* value		0.022*	
Pearson correlation		0.071	

Tests used: Independent t-test, ANOVA test, Pearson correlation, Tukey post hoc test

* Significant at level 0.05

### 3.4. Factors associated with perception toward the KD

Table 5 presents the factors associated with the perception toward the KD. Sex was significantly associated with perception score (*P* < .001), males had a significantly higher perception score compared to females (mean ± SD: 30.26 ± 4.92 vs 28.61 ± 4.72). Students had a significantly lower perception score compared to nonstudents (mean ± SD: 28.42 ± 4.71 vs 29.65 ± 5.06, *P* < .001).

**Table 5:** Factors associated with perception score toward KD in Saudi adults (*n* = 1071)

Factor	Perception score		*P* Value
	Mean	Standard deviation	
Sex			
Male	30.26	4.92	<0.001*
Female	28.31	4.72	
Student			
Yes	28.42	4.71	<0.001*
No	29.65	5.06	
University/college major			
Medical	27.12	4.73	<0.001*
nonmedical	29.02	4.58	
Course level			
Diploma	30.21	4.25	0.049*
Bachelor’s first	28.49	4.13	
Bachelor’s second and above	28.12	4.74	
Master	29.19	5.66	
PhD	29.77	4.44	
Have you followed a fad diet before? (a short-term weight-loss regimen like the cabbage diet, detox diet etc)?			
Yes	27.64	4.37	0.278
No	28.76	4.84	
Do you follow the ketogenic diet now?			
Yes	30.60	3.21	<0.001*
No	28.38	4.71	
No, but I have tried before	29.89	5.06	
Age correlation with Perception Score			
*P* value		<0.001*	
Pearson correlation		0.199	
BMI correlation with perception score			
*P* value		<0.001*	
Pearson correlation		0.112	

Tests used: Independent t-test, ANOVA test, Pearson correlation and Tukey post hoc test

* Significant at level 0.05

Students in medical majors had a lower perception score compared to nonmedical major students (mean ± SD: 27.12 ± 4.73 vs 29.02 ± 4.58, *P* < .001). Following a KD was also significantly associated with the perception toward a KD (*P* < .001). Tukey post hoc test revealed that those who previously followed a KD had a significantly higher perception compared to those who never did (*P* < .05). Both age and BMI had a significant positive weak correlation with perception score.

## 4. Discussion

To our knowledge, this is the first study to assess the usage of KD in a large Saudi population, compare between medical and nonmedical students and study the factors associated with the knowledge and perception of the KD. The results of this study showed that 24% of this Saudi population followed the KD. For those who currently/previously followed a KD (*n* = 253), *n* = 138/253 (54.5%) of the participants reported following it for 1–4 weeks. Forty-five percent of the participants strongly agree that the KD should only be recommended to individuals by a physician/dietitian and under their supervision. Half of the participants had low knowledge level (<50% of total score) (score of 8 and less) regarding the KD and 814 participants (76%) had moderate perception level. Students had a significantly lower mean knowledge score compared to nonstudents. Medical students had higher knowledge scores, but lower perception scores, compared to nonmedical students. Regarding KD perception, males had higher perception scores compared to females. Knowledge and perception scores were positively associated with BMI and age.

### 4.1. KD usage

In this study, 24% of the participants reported that they have previously followed the KD and only 0.5% were currently following the KD. This prevalence is not completely in line with a multinational study that included 17 Arabic countries and Saudi Arabia was 1 of them.^[17]^ Their results showed that 72% (from 1292 participants) were currently following the KD and 20% have previously followed the KD. The different results may be due to the variation in the statistical approach, the inclusion of 17 countries with different habits and cultures. Another explanation could be the difference in the mean BMI. This study had a mean BMI of 25.64 kg/m^2^ whereas Alhaj et al participants had a mean BMI of 29.9 kg/m^2^ indicating that the higher BMI may be a reason for the high percentage of participants currently following the KD.^[12]^ A recent study conducted in Jazan, Saudi Arabia in university students found 16.5% (from 701 students)^[16]^ followed the KD. Although the usage of KD varied between the studies, the prevalence is relatively high at both the national and international level which may be attributed to several factors. The majority of the participants in this study and other studies were females^[12,16–18]^ that may be more conscious in weight control and body shape. The KD imposes a restriction on a food category (i.e., carbohydrate) rather than quantity, and consequently leads to greater satiety time and less exposure to hunger, this may be an attribution to the high prevalence of KD usage.^[19]^

This study found that among all the users of the KD (*n* = 253), about 54.5% discontinued the diet within less than a month, while only 6.7% managed to stick to the diet for more than 6 months. These findings are inconsistent with the findings of a study conducted in Palestinian university students^[18]^ who found the average duration of KD use among the students at health sciences colleges to be 6 months. It could be that students of health-related majors have access to professional guidance and reliable information about the KD from their senior mates, mentors, and doctors, and therefore are expected to have a better commitment to the diet in comparison to their counterparts from the general population. Nevertheless, our findings are consistent with the results of D’Agostino^[12]^ and Alhaj et al^[17]^ in which the majority of participants (about 66%) were able to continue the diet for a period of time less than a month, yet only a minority (about 5%) reported following the diet for a period of time longer than 6 months. The duration of KD use was not assessed in the study conducted in Jazan, Saudi Arabia.^[16]^

Such poor compliance to the fad diets in general and the KD, in particular, was attributed in previous literature to the strict restrictions on food products that previously used to constitute the predominance of our diet including rice, bread, cereals, fruits, and all snacks containing added sugar.^[20,21]^ In fact, rice is 1 of the main components of the Saudi diet,^[22]^ which makes it difficult for some participants to comply with the KD diet for more than 6 months. Although not assessed in our study, we assume that the keto flu symptoms, which might manifest in the first few days upon initiating the diet are not easily bearable. Hence ketogenic dieters might find it difficult to keep up the restrictions, and eventually abandon the diet.^[23]^

### 4.2. Knowledge of the KD

Approximately half of the participants (49.5%) had low knowledge levels. These findings oppose the results of some studies.^[11,17]^ Alhaj et al^[17]^ found the overall knowledge level to be generally good among Arab adults in seventeen countries. Although their study had a larger number of participants than this study, no detailed information was provided on how many Saudi adults were in the study. Furthermore, in Pakistan, students had good knowledge of the KD.^[11]^ However, they were medical students which may be an explanation for the different results. Our results are in accordance with several studies in the US,^[12]^ Palestine^[18]^ and Saudi students^[16]^ who attributed the poor knowledge level of participants to the unreliable sources that people usually gain information from such as social media.

### 4.3. Perception of the KD

Meanwhile, in the field of perception, we found that 76% had a moderate level. Likewise, the moderate level of perception in this study corresponds to the study of D’Agostino whose sample had in general neutral level of perception.^[12]^ This can possibly be explained by the popularity of the KD nowadays, making people share their ideas and understanding of the KD with each other, leading finally to similar perception levels. Unfortunately, not only would poor knowledge and perception level of the KD threaten the success of dieters’ attempts to lose weight, but also jeopardize their health. For example, when fat is exclusively acquired from unhealthy sources like red meat, deep-fried food, and processed food rather than healthy sources like olive oil, seeds, fish, and nuts. It is therefore of substantial importance to explore and subsequently address the reasons behind people poor understanding of the KD.

### 4.4. Factors associated with knowledge and perception of KD

In this study, no significant association between sex and knowledge score level was noted, supporting the findings of Alhaj et al^[17]^ who found similar findings. However, a significant association between the male sex and high perception level was observed. These findings partly correspond to the study of D’Agostino^[12]^ where the male sex was associated with significantly higher scores in both knowledge and perception. This correlation is possibly related to the nature of fat distribution among males, which tends to accumulate around the abdomen rather than hips, taking the shape of an apple, eventually predisposing the individual to obesity-related complications like type 2 diabetes and producing cosmetically disfiguring contour of abdomen; hence men seek and subsequently read and learn about means of weight loss like fad diets more frequently than women, whose obesity are pear-shaped, associated with less complications, and fewer cosmetic issues.^[24]^

Unexpectedly, a significant association between being a student and having significantly lower knowledge and perception scores was found in this study. This supposedly is justified by the fact that students oftentimes are busy with their studies, leaving less time for them to read and learn.^[25]^ Moreover, young people are less experienced and not much exposed to the topic of nutrition and diet compared to their older counterparts. Studying a master degree or diploma has also been significantly associated with higher knowledge scores & perception levels respectively. These findings correspond to the study of Alhaj et al^[17]^ who found that knowledge scores to be significantly higher in participants with a diploma or higher degree of education. This is because, with a higher degree of education, people become more professional and systematic at pursuing authentic information, from which the foundation of knowledge and perception is then made.^[26]^

We found in our study that with increasing weight, the knowledge and perception scores get higher. These findings are consistent with the study of Altamimi et al^[18]^ who also found the knowledge scores to be highest among obese participants. However, it opposed the findings of Alhaj et al^[17]^ who noted in their study a significant association between lower BMI and higher score of knowledge. We assume that with a higher index of body mass, people start to be concerned more about their shape and health, looking for and learning about trending strategies of healthy weight loss in pursuit of higher satisfaction and longer life.^[27]^ In our study, increasing age was also a factor associated with higher scores of knowledge and perceptions. This is in contrast to the study of Alhaj et al^[17]^ who found the greatest score of knowledge to be among middle-aged participants. We hypothesize that older age participants have their health progressively getting poorer and thus might have been counseled before to oppose that by following a healthy lifestyle through diet and physical activity, resulting ultimately in higher score of knowledge and perception.

We found in our study that having a past experience with fad diets is associated significantly with higher scores of knowledge and perception about the KD whereas following a KD at the present time was found to have a significant association with higher scores of knowledge. These findings support the study of Altamimi et al^[18]^ where the users of the KD were found to have significantly higher scores of knowledge and perception. Furthermore, they agree with the results of D’Agostino^[12]^ who attributed such correlation to either personal interests or self-learning prior to going through a new experience of weight loss. On top of that, we assume that people, who are willing to lose weight through fad diets like the KD, are likely to seek help from dieters who had past successful attempts of weight loss, providing them with a comprehensive experience that eventually improves their overall awareness.

### 4.5. Comparison between medical and nonmedical students

D’Agostino^[12]^ stated in the study that students with medical majors had a higher score of knowledge but lower score in perception compared to their counterparts with nonmedical majors, proposing a significant association between medical majors and the level of knowledge and perception. These findings are in agreement with our study, in which students with medical majors were found to have a higher score of knowledge but lower perception level. Apparently, as suggested by D’Agostino, this correlation is secondary to the fact that students with medical majors are obligately engaged in heath and nutrition-related courses like biochemistry which aids in comprehending the basis of the KD. Moreover, we assume that students with medical majors are naturally more interested in fields closely related to their specialties like nutrition, and thus are expected to self-learn and consequently have better knowledge about fad diets like the KD. We also believe that students who never followed or discontinued KD is the reason for the lower score of perception and this could be referred to the reason where people believe that KD is not safe for long-term use as it could expose users for nutrition deficiencies.^[28]^

### 4.6. Public health implications

There are several ways in which this study may be used for educational purposes or to increase the awareness of population. Dieticians may measure the knowledge and perception of the KD diet in patients who visit the outpatient clinics to lose weight. If knowledge and perceptions are low, dietitians can increase their knowledge through education. Another suggestion is that universities and colleges should aim to increase students’ knowledge and perception of the KD in all majors even for medical field students. In addition, clinical nutrition programs can make awareness campaigns regarding the KD including their side effects, advantages and people who are not recommended to follow it. The significant factors associated with knowledge and perception of the KD could be considered in campaigns and education sessions. For example, higher educated people can receive less information than less educated people. We believe that social media may also be used in public education regarding the KD, where the information used for this purpose, should be given under the supervision of dietitians and/or public health specialists. Finally, future studies examining the efficacy of the KD in the setting of optimized circumstances such as using a standard KD among all users are needed to provide a final judgment of the KD as a strategy for weight loss.

### 4.7. Strengths and limitations

To our knowledge, this is the first study conducted in a Saudi population and included a large number of participants (*n* = 1071). While the generalizability of our findings to the entire population may be limited, our study contributes valuable insights and knowledge within the specific context and population of interest. In addition, A prevalidated tool was used to assess the knowledge and perception of the KD. Furthermore, this is the first study that assessed the factors associated with the knowledge and perception of participants to the KD diet and compared between medical and nonmedical Saudi students. In this study, there are limitations that can be addressed in future research. First off, the data were collected via a self-administered questionnaire disseminated through social media. This could have yielded less accurate findings as surveys received this way usually are taken lightly and filled in a hurry by the participants. Secondly, most of our respondents are females (77.7%), larger studies with equal number of males and females representing the Saudi population are needed. Finally, most participants (54.5%) who used the KD reported following the diet for less than a month, possibly yielding less accurate results in regard to the efficacy of the KD.

## 5. Conclusion

About 24% of this Saudi study population followed the KD. Half of our sample had a low knowledge level and 76% had moderate levels of perception regarding the KD. Medical students had higher knowledge scores, but lower perception scores, compared to nonmedical students. Knowledge and perception scores were positively associated with BMI and age. This study highlights the need for education on the KD diet in Saudi adults. The factors associated with KD knowledge and perception found in this study may be beneficial when designing future experimental studies and ultimately, contribute to better healthcare outcomes and improved management of obesity.

## Author contributions

Data curation: Ameerah A. Alhassani, Essra A. Noorwali.

Investigation: Ameerah A. Alhassani, Essra A. Noorwali.

Writing – original draft: Ameerah A. Alhassani.

Conceptualization: Essra A. Noorwali.

Formal analysis: Essra A. Noorwali, Ameerah A. Alhassani.

Methodology: Essra A. Noorwali, Ameerah A. Alhassani.

Project administration: Essra A. Noorwali.

Supervision: Essra A. Noorwali.

Visualization: Essra A. Noorwali.

Writing – review & editing: Essra A Noorwali.

## Supplementary Material




