# Supplementary material for: Usage, knowledge and perception of the ketogenic diet and associated factors in Saudi adults: A cross-sectional study
Source: Medicine (Baltimore). 2024 Feb 9;103(6):e37063. doi: 10.1097/MD.0000000000037063 (PMC10860923; doi:10.1097/MD.0000000000037063)
Supplement: Supplementary file 1 [file medi-103-e37063-s001.docx]

**Supplementary material 1 - Consent form**

Knowledge, perception, and use of ketogenic diet in Saudi population

By completing this questionnaire, I agree to participate in this study. Before the last answer, I can freely and without any consequences terminate my participation.

Consent Form

This questionnaire aims to measure knowledge, perception, and use of the ketogenic diet in Saudi population. The study is conducted by Ameerah Ahmad Al-Hassani, under the supervision of Dr. Essra Noorwali for the purpose of a master's degree in clinical nutrition at Umm Al-Qura University.

The study takes about 15 minutes to complete and is strictly private. Participants must first complete a set of demographic information. They would next be asked a series of broad questions on their weight loss experiences. Following this, they will answer many questions about knowledge on the ketogenic diet to the best of their abilities. In the final segment, participants will be asked perception questions and their feelings towards the ketogenic diet will be assessed.

All responses are regarded as confidential, and individual participants' responses will never be identifiable. Rather, all data will be pooled, and only aggregated data will be presented.

Thanks for your contribution and good cooperation. For further information please contact: eanoorwali@uqu.edu.sa \ ameerah_ahmad@hotmail.com
