# Supplementary material for: Usage, knowledge and perception of the ketogenic diet and associated factors in Saudi adults: A cross-sectional study
Source: Medicine (Baltimore). 2024 Feb 9;103(6):e37063. doi: 10.1097/MD.0000000000037063 (PMC10860923; doi:10.1097/MD.0000000000037063)
Supplement: Supplementary file 2 [file medi-103-e37063-s002.docx]

**Supplementary material 2 -categorization of medical and non-medical fields based on majors in Medical or Health-Related Colleges.**

**Arts and Sciences College**

- Sciences of Biology (cell biology and molecular genetics, ecology and evolutionary biology, integrative physiology and neurobiology, biology)
- Chemical science (all chemistry including biochemistry)
- Scientific Research in Biomedical Sciences (neuroscience, physiology, pharmacology, clinical psychology, clinical epidemiology)
- Anthropology of Medicine -Pre-Medical -Pre-Pharmaceutical Physiological Sciences -Physical Science -Physics

**Education, Health, and Human Services College**

- Mental Health Clinics Counseling
- Sciences of Education (life science, life science/chemistry) -Exercise Physiology -Exercise Science
- Health Promotion and Education
- Sciences of Health
- Hospitality Administration
- Family Studies and Human Development
- Sexuality in Humans
- Studies on Integrated Health
- Nutrition
- Physical Therapy

All other majors were categorized as non-medical. Examples of non-medical majors

- Business
- Administration
- Art
- Geography
- Languages
- Mathematics
- Accounting
- Engineering
